# A Rare Ocular Manifestation of Idiopathic Hypertrophic Cranial Pachymeningitis

**DOI:** 10.7759/cureus.20633

**Published:** 2021-12-23

**Authors:** Josephine En Hui Lee, Suresh Subramaniam, Chun Fai Cheah, Kok Hoe Chan, Hussein Adil

**Affiliations:** 1 Ophthalmology and Visual Sciences, School of Medical Sciences, Universiti Sains Malaysia, Kota Bharu, MYS; 2 Ophthalmology, Hospital Raja Permaisuri Bainun, Ipoh, MYS; 3 Neurology, Hospital Raja Permaisuri Bainun, Ipoh, MYS; 4 Radiology, Hospital Raja Permaisuri Bainun, Ipoh, MYS

**Keywords:** superior ophthalmic veins dilatations, anterior scleritis, anterior uveitis, cranial nerve palsy, chronic headache, idiopathic hypertrophic cranial pachymeningitis

## Abstract

Idiopathic hypertrophic cranial pachymeningitis (IHCP) is a rare form of thickening of the dura mater. There are limited reports on the ocular manifestation of IHCP and its treatment. Up to our knowledge, there is no report on bilateral superior ophthalmic veins (SOV) dilatation with IHCP and there are only a few reports on anterior scleritis with IHCP. We report a 62-year-old gentleman with underlying hypertension and chronic headache who presented with fever, headache, and unresolving both eyes redness as manifestations of bilateral anterior scleritis, anterior uveitis, secondary glaucoma, and multiple cranial nerve palsies. Magnetic resonance imaging of the brain showed global thickening and enhancement of the pachymeninges with bilateral SOV dilatations. The diagnosis of IHCP was made after ruling out infective and autoimmune causes. The patient was treated with oral prednisolone, oral azathioprine, topical timolol maleate, topical dexamethasone, and topical moxifloxacin. The patient was successfully treated and was stable throughout two years review. In conclusion, unresolved red eyes with headaches can be an early presentation of IHCP. Pathophysiology and treatment of the ocular manifestations and IHCP were discussed.

## Introduction

Hypertrophic pachymeningitis is a thickening of the dura mater caused by diffuse inflammatory diseases. It can be divided into "primary," where there is no identifiable cause found, and "secondary," where there is an identifiable cause [1]. Secondary causes can be malignancy, infection, and autoimmune processes. When the evaluation fails to reveal a cause, a diagnosis of idiopathic hypertrophic cranial pachymeningitis (IHCP) is made [2]. IHCP may have different clinical presentations. We report a rare case of IHCP with the presentations of bilateral anterior uveitis, anterior scleritis, secondary glaucoma, multiple cranial nerves (CNs) palsies, and bilateral superior ophthalmic veins (SOVs) dilatation. Up to our knowledge, there is no report on bilateral SOVs dilatations with IHCP.

## Case presentation

**Figure 1:**
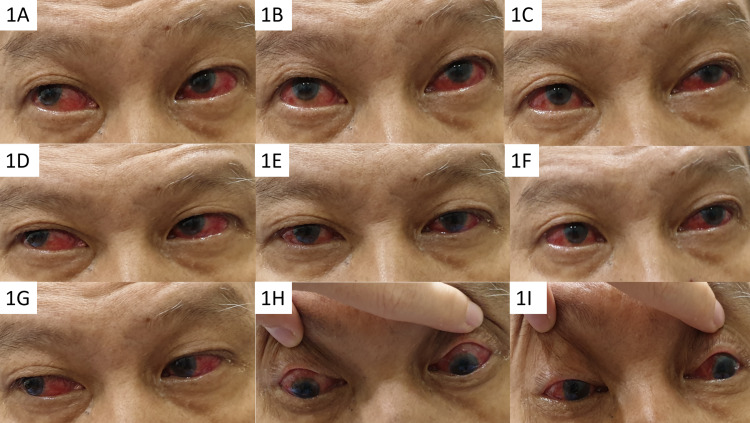
Clinical image showing nine cardinal gazes (1A-1I). Primary gaze (1E) showed orthophoria. There were restricted eye movements in all gazes (1A-1I except 1E).

**Figure 2:**
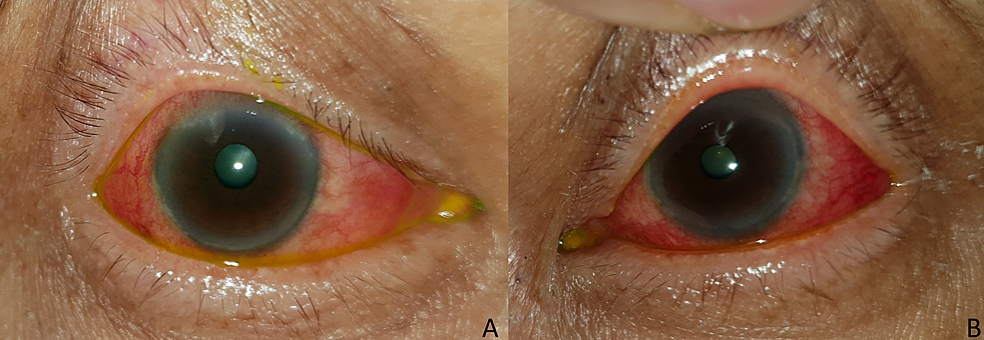
Right (A) and left (B) eyes showed diffused conjunctival injection.

A 62-year-old gentleman with underlying hypertension and a chronic headache was admitted to the medical ward for worsening of his headache for four days, associated with fever, vomiting, and giddiness. He was referred to the ophthalmology team for persistent bilateral eyes redness despite the use of topical antibiotics for a one-week duration. He denied blurring of vision, eye pain, photophobia, or diplopia. Initial ocular examination was normal aside from bilateral generalized conjunctival injection. One week later, the intraocular pressure (IOP) was raised to 30 mmHg in the right eye and 34 mmHg in the left eye with no evidence of uveitis or angle closure. The fundus examinations were normal. He was started on topical timolol maleate twice a day. Two weeks after the initial onset of symptoms, he developed third, fourth, sixth, and seventh lower motor neurons, and eight CNs palsies (Figure 1). Ocular examination revealed bilateral diffuse conjunctival injections, which did not blanch with phenylephrine, along with features of bilateral non-granulomatous anterior uveitis (Figure 2). Bilateral ocular ultrasound showed thickened scleral with no T-sign. Both eyes were managed with topical timolol maleate twice a day, topical dexamethasone four times a day, and topical moxifloxacin four hourly.

His blood investigations showed raised C-reactive protein (265.4 mg/L), erythrocyte sedimentation rate (119 mm/hr), lactate dehydrogenase (203 u/L), and cancer antigen 125 (41.5 u/ml). Blood culture and sensitivity yield no growth, while infective screenings were non-reactive. No clinical evidence of connective tissue disease was found. The perinuclear antinuclear cytoplasmic antibody, antineutrophil cytoplasmic antibody, antinuclear antibody, complement C3, and complement C4 were all negative. Serum angiotensin-converting enzyme test was not done due to financial constrain. No clinical signs and symptoms were suggestive of sarcoidosis. The colonoscopy showed no abnormality.

**Figure 3:**
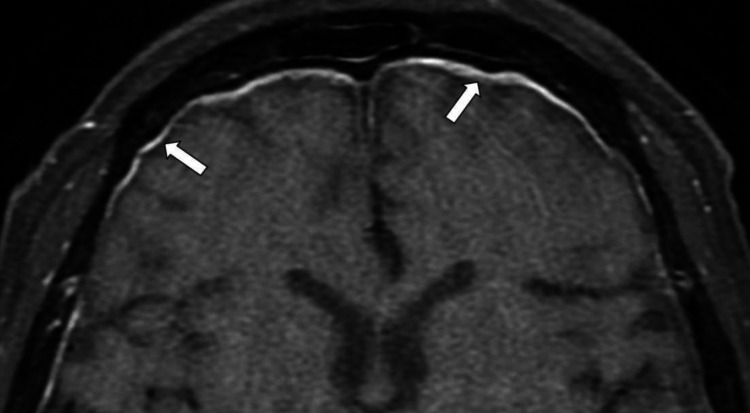
Axial T1-weighted (fat suppression) post-contrasted MRI of the brain. MRI of the brain showed thickened and enhancing pachymeninges (white arrows).

**Figure 4:**
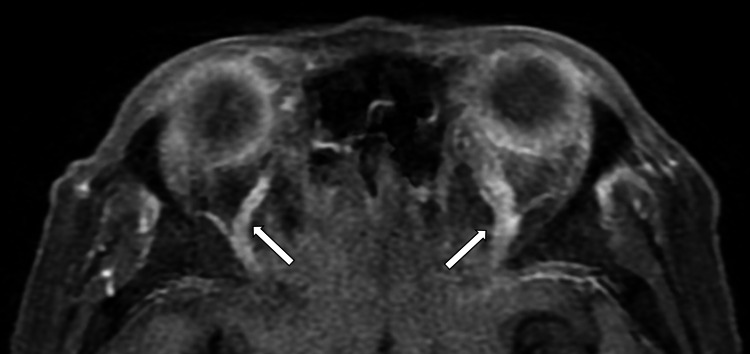
Axial T1-weighted (fat suppression) post-contrasted MRI orbit image. MRI orbit image showed the dilated left and right superior ophthalmic veins (white arrows). The bilateral superior ophthalmic veins were opacified by contrast with no filling defect seen within.

**Figure 5:**
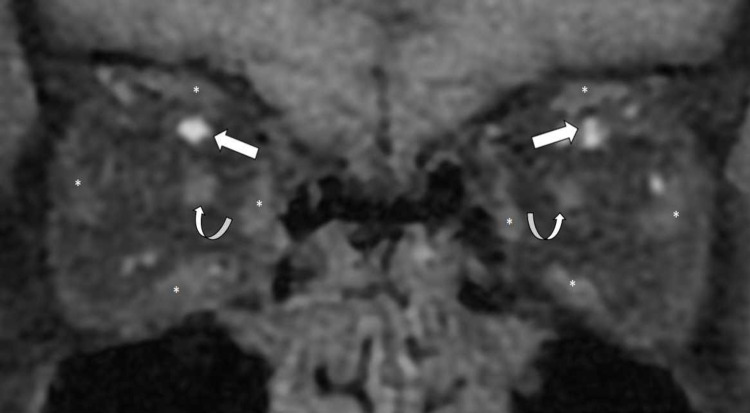
Coronal T1-weighted (fat suppression) post-contrasted MRI orbit image. MRI orbit image showing the dilated left and right superior ophthalmic veins (white arrows), the optic nerves (curved white arrows), and extraocular muscle (*).

Magnetic resonance imaging (MRI) of the brain reviewed generalized thickened and enhancing pachymeninges (Figure 3). There were asymmetrical bilateral SOVs dilatations (5.7 mm for the right eye and 3.4 mm for the left eye) with no thrombosis seen (Figures 4, 5). The cavernous sinus appeared to be intact. There were no obvious changes of CN from the imaging. No aneurysm or arteriovenous malformation was found. MRI orbit, ultrasound abdomen, and computed tomography of thorax, abdomen, and pelvis were unremarkable. Cerebrospinal fluid (CSF) analysis showed a very high protein level of 1729 mg/L. CSF analysis for infective screening was reported negative.

**Figure 6:**
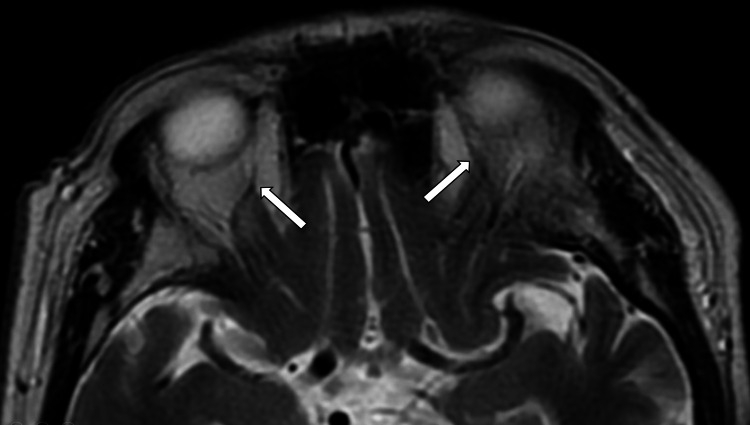
Axial T2-weighted MRI orbit post-treatment. Post-treatment MRI orbit image showed the left and right superior ophthalmic veins (white arrows) return to the normal caliber.

Diagnosis of IHCP with bilateral non-necrotizing diffused anterior scleritis, non-granulomatous anterior uveitis with secondary glaucoma, and multiple CNs palsies was made. The patient was started on oral prednisolone 30 mg once per day (0.5 mg/kg/day) and oral azathioprine 25 mg once per day, which tapered up slowly to 100 mg once per day (2 mg/kg/day). Fever, headache, and lower motor neuron seventh CN palsy resolved while eight CNs palsies persist. Bilateral anterior uveitis and scleritis were resolved after one month of topical dexamethasone. Both eyes’ IOP were normalized during the third-month follow-up and the patient was medication-free. Third, fourth, and sixth CNs palsies were resolved after six months. Repeated MRI of the brain at 15 months reviewed absence of bilateral dilated SOVs (Figure 6). The patient was on a maintenance dose of oral prednisolone 5 mg once per day and oral azathioprine 100 mg once per day. There was no recurrence of disease within two years of follow-up.

## Discussion

Pachymeningitis is defined as diffuse or focal dura mater thickening on MRI of the brain and/or persistent inflammation in the histologic analysis of dura mater [3]. CNs, cavernous sinus, and the optic nerve sheath are encased by the dura mater, thus they are prone to get involved in pachymeningitis [4]. IHCP usually presents with headaches and CN palsy [3,5]. Other presentations include orbital pseudotumor, uveitis, and cavernous venous thrombosis [3].

Of patients with IHCP, 17% can present with uveitis [3]. Yaylali et al. reported a case on IHCP, which presented with intermediate uveitis. They postulated the cause of the IHCP to be autoimmune in nature [2]. There are only a few reports on anterior scleritis as the initial presentation of IHCP [6,7]. In our patient, the only positive investigations results were the inflammatory markers. Therefore, similarly to Yaylali et al., we too attribute the pathogenesis of anterior uveitis and scleritis in this patient to autoimmune mechanisms. Inflammatory leukocytes in the anterior chamber obstruct the trabecular meshwork most likely caused by the secondary raised IOP. This is evidenced by the resolution of manifestations after the commencement of oral prednisolone and oral azathioprine. The prognosis is good as there was no recurrence within two years.

The commonly affected CNs in hypertrophic pachymeningitis are the second and seventh CNs. Third, fourth, and sixth CNs involvement have been reported as well [4]. The CNs involvement in our patient may be due to infiltration of the dura that surrounds the CNs as part of the autoimmune processes. Complete resolution of ophthalmoplegia was achieved after six months in this patient. This is also observed by Mekinian et al. in their retrospective study too [3].

There are various causes of dilated SOV. It can be divided into three main groups, which are venous thrombosis, inflammatory, and vascular malformation [8]. The mechanism of action can be an impaired venous outflow with congestion from intraluminal obstruction, mass or local inflammatory process compressing on SOV externally, and orbital vascular hydrodynamic changes [8]. Compression of cortical vessels and inflammatory infiltration into brain parenchyma could lead to venous congestion and ischemia of the parenchymal [4]. The pathophysiology of the bilateral dilated SOVs in this patient can be due to thickened meninges causing venous congestion. These were resolved after treatment of IHCP.

The first line of systemic treatment for IHCP is immunosuppressant such as corticosteroid (1 mg/kg/day). Azathioprine (2-3 mg/kg/day), cyclophosphamide, rituximab, and methotrexate are the other options when disease persists or recurs during corticosteroid use [4]. The patient’s ocular manifestations resolved within six months with azathioprine, systemic, and topical corticosteroid. The prognosis of IHCP varies. Early treatment reduces neurological and ocular deficits. Intravenous methylprednisolone was shown to improve neurological deficits and decrease recurrence of pain [5]. Neuralgia may be improved with oxcarbazepine [5]. Maintenance of steroids and azathioprine is important to prevent recurrences.

## Conclusions

Unresolved red eyes with headaches can be an early presentation of IHCP. These symptoms should raise suspicion of a general practitioner for an early referral and workup. Close follow-up is crucial as other signs such as raised IOP and CN palsies can present later. Bilateral SOVs dilatations could be part of IHCP manifestations. Diagnosis of IHCP is challenging. Thorough investigations including laboratory and imaging are necessary to rule out life-threatening causes. Prompt treatment with immunosuppressive drugs is important to achieve a good prognosis.
